# Mitochondrial DNA Suggests the Introduction of Honeybees of African Ancestry to East-Central Europe

**DOI:** 10.3390/insects12050410

**Published:** 2021-05-02

**Authors:** Andrzej Oleksa, Szilvia Kusza, Adam Tofilski

**Affiliations:** 1 Department of Genetics, Faculty of Biological Sciences, Kazimierz Wielki University, Powstańców Wielkopolskich 10, 85-090 Bydgoszcz, Poland; 2 Centre for Agricultural Genomics and Biotechnology, University of Debrecen, 4032 Debrecen, Hungary; kusza@agr.unideb.hu; 3 Department of Zoology and Animal Welfare, University of Agriculture in Krakow, Adama Mickiewicza 24/28, 30-059 Kraków, Poland

**Keywords:** COI-COII intergenic region, hybridisation, African haplotypes, Europe

## Abstract

**Simple Summary:**

In Europe, a well-known threat to the conservation of honeybee diversity is the loss of genetic uniqueness of local populations due to beekeepers’ preference for a few genetic lineages. However, due to climate change and large-scale ongoing movement of breeding individuals, the expansion of bees of African origin could represent another threat. This issue has not yet been recognised in detail, although bees bearing African mitochondrial DNA occur in South-West and South Europe due to natural gene flow. Here, we determine the diversity of mitochondrial DNA in honey bees from East-Central Europe. We sequenced the COI-COII region in 427 bees sampled along two 900 km transects (17.5° N and 23° E). We found that 1.64% of bees (95% CI: 0.66–3.35%) had African mitochondrial DNA. It is unlikely that their presence in the area resulted from natural migration but instead human-driven introductions of hybrids of African ancestry. This expansion deserves more attention, as it may contribute to the dissemination of undesirable traits, parasites and diseases.

**Abstract:**

In Europe, protecting the genetic diversity of *Apis mellifera* is usually perceived in the context of limiting the spread of the evolutionary C-lineage within the original range of the M-lineage. However, due to climate change and large-scale ongoing movement of breeding individuals, the expansion of bees from the African A-lineage could represent another threat. This issue has not yet been investigated in detail, although A-mitotypes occur in South-West and South Europe due to natural gene flow. Here, we determine the diversity of mtDNA in honey bees from East-Central Europe. We sequenced the COI-COII region in 427 bees sampled along two 900 km transects (17.5° N and 23° E). We found that 1.64% of bees (95% CI: 0.66–3.35 %) had A-mitotypes. It is unlikely that their presence in the area resulted from natural migration but instead human driven introductions of hybrids of African ancestry. This expansion deserves more attention, as it may contribute to the dissemination of undesirable traits, parasites and diseases.

## 1. Introduction

There is a growing concern that the genetic variability of the western honey bee, *Apis mellifera*, is under serious threat due to human activities. Some studies have suggested that this reduced variability may contribute to the worrying global decline of bees [1,2,3]. In general, a decrease in genetic variation may result from two types of mechanisms. Firstly, selective breeding usually leads to reduced genetic diversity in domesticated animals [4,5]. Secondly, the human-mediated transportation of bees (including transhumance of colonies and trade of queen bees) may result in the loss of differences between locally adapted populations–in other words, a decrease in between-population variation [6], despite this potentially being accompanied by an increase of within-population genetic diversity [7]. As a result, many local varieties (subspecies and ecotypes) are on the path towards loss of genetic uniqueness due to replacement or hybridisation with introduced bees [8,9,10,11,12]. Because admixture is an increasingly severe threat to maintaining the diversity of honey bees, the need to better understand the spread of non-native subspecies of *A. mellifera* is gaining in urgency.

The global diversity of *A. mellifera* is well recognised, as the honey bee has been the subject of numerous population genetic surveys using maternal and biparental genetic markers [13,14,15,16,17,18], along with morphometry [19,20,21,22,23]. These studies reveal high diversification within the species, with approximately 30 described subspecies grouped into five major evolutionary lineages: A (African), C (subspecies from east and south of the Alps), M (subspecies ranging from western and northern Europe to central Asia in the east), O (Oriental, subspecies from the Middle East), Y (subspecies from Arabian Peninsula and Ethiopia) [15,17,22,24,25]. Since the natural range of the honey bee covers diverse environments, from tropical to temperate biomes [22,26], local populations of the species have adapted to unique ecological conditions. For that reason, indigenous bee populations are essential reservoirs of local adaptations [10,27]. Bee colonies of local provenance survive for longer in their original environmental conditions than imported bees [28]. Despite this, in Europe and worldwide, most honey bee queen breeders use only two of more than thirty subspecies: the Italian *A. m. ligustica,* and the Carniolan honey bee *A. m. carnica*, which are among the favourite subspecies kept by beekeepers [1]. Both belong to the evolutionary C-lineage, whose natural distribution encompasses Southeastern Europe, including Italy and the Balkans, and the adjacent part of Central Europe. Italian and Carniolan queens are massively imported to areas originally inhabited by other subspecies. In vast parts of Europe, this has resulted in the replacement or loss of genetic uniqueness of the European dark bee *A. m. mellifera* [8,9,10].

Aside from the massive importation of bees of the C-lineage, another phenomenon strongly affecting the honey bee’s genetic distribution is the spread of genes of African origin. The best described example of this is the expansion of ‘Africanised’ bees within the New World, where African bees (*A. m. scutellata*) were introduced and created highly invasive hybrids with bees of European origin such as the European dark bee *A. m. mellifera,* the Italian bee *A. m. ligustica* and the Iberian bee *A. m. iberiensis* [29]. Elsewhere, bees with mitochondrial haplotypes typical of the A-lineage spread through Southern Europe as a result of natural migration events. Outside of Africa, bees with A-lineage haplotypes have also been reported in the Iberian Peninsula [30,31,32,33,34,35], the adjacent area of Gascony in South-West France [36], Mediterranean islands (Sicily [37], Malta [38], Baleares [39]) and Macaronesia [40,41]. We are not aware of any reports of the presence of African mitotypes elsewhere in Europe or in the Middle East.

In this study, we aimed to investigate the mitochondrial DNA diversity of honey bees in East-Central Europe. In this part of the continent, the Carpathian mountain range has been the main biogeographical barrier for many organisms. These mountains have also constituted a putative border between the M- and C-lineages, i.e., between West- and North-European dark bees (*A. m. mellifera*) and Carniolan bees (*A. m. carnica*) from the Pannonian Basin and adjacent areas [22]. However, in recent decades, the border between these evolutionary lineages has become blurred, as beekeepers have increasingly preferred the latter subspecies. Significant numbers of Carniolan queens are imported north of the Carpathians, changing the mitochondrial gene pool of the honey bee population [42]. *A. m. mellifera* does not enjoy similar popularity among beekeepers [43], and is not intensively traded, so we did not expect it to occur frequently in the native area of *A. m. carnica*. Thus, we hypothesised that (1) bees in East-Central Europe typically have mitotypes derived from the C- or M-lineages, (2) in the area north of the Carpathians, native honey bees of the M-lineage have to a large extent been replaced by the imported C-lineage, and (3) the frequency of M-lineage mtDNA is increasing northwards. Surprisingly, we found evidence that almost 2% of bees in East-Central Europe can be considered African by matrilineal descent.

## 2. Materials and Methods

**Figure 1:**
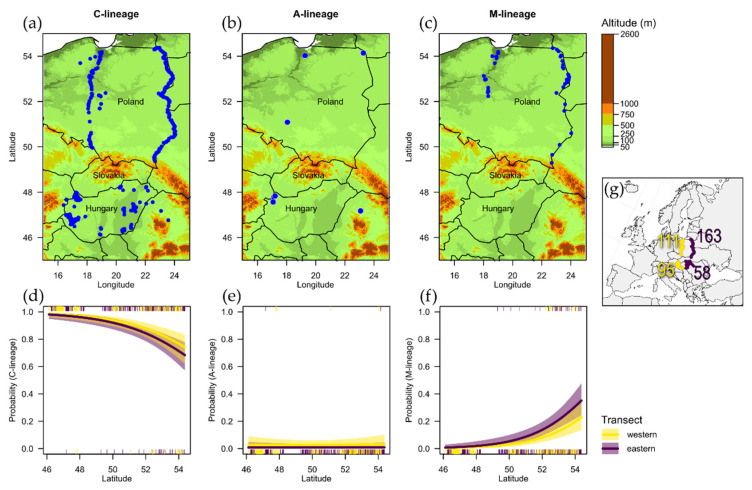
(**a**–**c**) Distribution of bees with mtDNA from C-, A- and M-lineages in the studied area and (**d**–**f**) logistic regression curves showing relationships between mitotypes occurrence and latitude. (**g**) General location of the study area in Europe; numbers indicate the sample sizes obtained from different parts of study area (western and eastern transect, i.e., west or east to 20° E meridian; north and south of the Carpathians).

We investigated the maternal origin of honey bees in Central-Eastern Europe, considering an area encompassing northern Poland, Hungary and Romania (Figure 1g). The northern part of the study area (most of Poland) is part of the North European Lowland, while the southern zone (the Carpathians and Pannonian Basin) covers the Alpine system of young mountains and basins north of the Mediterranean. The Carpathians were the most likely geographical barrier to admixture between *A. m. mellifera* in the north and *A. m. carnica* in the south, and the exact location of the hybridization zone between subspecies was not thoroughly investigated before human activity blurred the boundaries of these subspecies [22,44,45,46].

For this study, we sampled bees on both sides of Carpathians (n = 153 on the southern side and n = 274 on the northern side of the mountain range, Figure 1g). Foraging worker bees were captured with an entomological net from flowers in 2017–2018, immediately preserved in 90% ethanol, and stored in a −20 °C freezer until DNA extraction. Samples were collected along two latitudinal transects in Poland: (1) along the eastern border of the country, approximately along the 18° E meridian (N = 163) and (2) in the middle of Poland, roughly along the 23° E meridian (N = 111). Samples from Hungary, together with a few bees from the adjacent part of Romania, were collected from more dispersed random locations, N = 58 and N = 95 from the east and west, respectively (Figure 1). Overall we believe that both sampling designs (linear and scattered) were equally efficient in providing us with an adequate representation of bees living in the study area.

Genomic DNA was extracted from the bee thoraces using the Insect Easy DNA Kit (EZNA) (Omega Bio-Tek, Norcross, GA, USA), according to the manufacturer’s recommended procedure.

From each location in Hungary, only one worker was sampled, while in Poland up to 4 workers per site were sampled and genotyped. However, the distances between neighbouring sampling sites in Poland were greater than in Hungary (median distance between closest sampling sites was 4.4 km in Poland and 2.4 km in Hungary). To make sure that bees were unrelated and represented independent data points, we genotyped them using 13 microsatellite loci (detailed information on loci used in [47]), estimated relatedness among pairs of individuals sampled at distance <5 km (Queller–Goodnight estimator, computed with R-package ‘related’ ver. 0.8 [48]) and left only single individuals from each pair sharing the same mtDNA haplotype and relatedness significantly exceeding zero (lower 9% confidence interval of *r* > 0).

**Figure 2:**
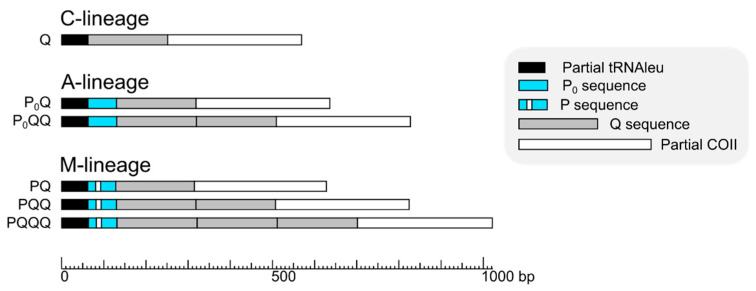
Schematic representation of COI-COII intergenic region of the mtDNA examined in this study. It is composed of partial sequences of tRNAleu and COII genes, and a variable number of non-coding sequences P and Q. Bees of C-lineage have only one Q-sequence and no P-sequences, while A- and M-lineages have up to three Q-sequences and one P-sequence, which occurs in two variants, i.e., P_0_ in A-lineage, and P in M-lineage.

Samples were analysed to specifically genotype the mitochondrial region located between the tRNA^leu^ and COII genes (originally named COI-COII intergenic region) [49,50]. PCR amplification of the sequence was performed using E2 (5′-GGCAGAATAAGTGCATTG-3′) and H2 (5′-CAATATCATTGATGACC-3′) primers following the method described in [51]. We chose the COI-COII region due to its extensive prior use in studies of *A. mellifera*. Its amplification, followed by restriction with *Dra*I enzyme, has been used since the 1990s as the most popular PCR-RFLP assay to discriminate the maternal origin of honey bees [51,52]. The COI-COII region has variability which results from the presence or lack of two types of repeated, non-coding sequences, named P and Q (Figure 2).

The PCR reaction was set up in a 50 μL volume, containing 25 μL 2× Multiplex PCR Master Mix (QIAGEN, Hilden, Germany), 1 μM of each primer, 5 μL (10 ng/μL) of the template DNA and ddH_2_0 to complete the final volume. Amplifications were conducted with PTC200 thermal cycler (MJ Research, Waltham, MA, USA) using the following profile: initial denaturation at 95 °C for 15 min, followed by 29 cycles of {a denaturation step at 94 °C for 30 s, an annealing step at 50 °C for 45 s, and an extension step at 64 °C for 1 min}, and a final extension step at 72 °C for 10 min.

The PCR products were purified using a PCR/DNA Clean-Up Purification Kit (EURx) and then sequenced bidirectionally using the Sanger method on an ABI PRISM^®^ 3100 Genetic Analyzer (Applied Biosystems). Forward and reverse reads were assembled into contigs using BioEdit version 7.2.5 [53]. The sequences were aligned with the MAFFT online service version 7 at https://mafft.cbrc.jp, accessed on 20 March 2021, using the FFT-NS-i algorithm [54].

The number of unique mitochondrial haplotypes (mitotypes), their frequency and genealogical relationships (based on a statistical parsimony haplotype network with a 95% connection limit and simple indel coding method [55]) were estimated using the R package ‘mitotypes’ v.1.0 [56]. Additionally, we constructed maximum-likelihood trees using MEGA X [57]. Initial trees for the heuristic search were obtained automatically by applying Neighbor-Join and BioNJ algorithms to a matrix of pairwise distances estimated using the Maximum Composite Likelihood (MCL) approach, and then selecting the topology with superior log likelihood value. The substitution model (Hasegawa-Kishino-Yano model, HKY [58]) applied in the analysis was selected on the basis of the Akaike information criterion with IQ-TREE 2 [59].

95% confidence limits around observed proportions of mitotypes were calculated using ‘binom.confint’ function with methods = ‘exact’ from the R package ‘binom’ [60].

The obtained sequences were compared with the GenBank database using the online version of BLAST (available at http://blast.ncbi.nlm.nih.gov, accessed on 20 March 2021). Unique mitotypes were submitted to GenBank under accession numbers MW939577-MW939620.

The relationship between the presence of a particular mitotype and geographic coordinates was explored using logistic regression (i.e., generalised linear models with a logit link function). In models, we coded mitotypes as binary variables (for example, A-lineage mitotype or non-A-lineage mitotype). Because we did not have observations for the full range of longitude, we included this variable as a categorical variable (‘transect’) with two levels (with 20° E as the demarcation value for samples from west and east).

## 3. Results

**Figure 3:**
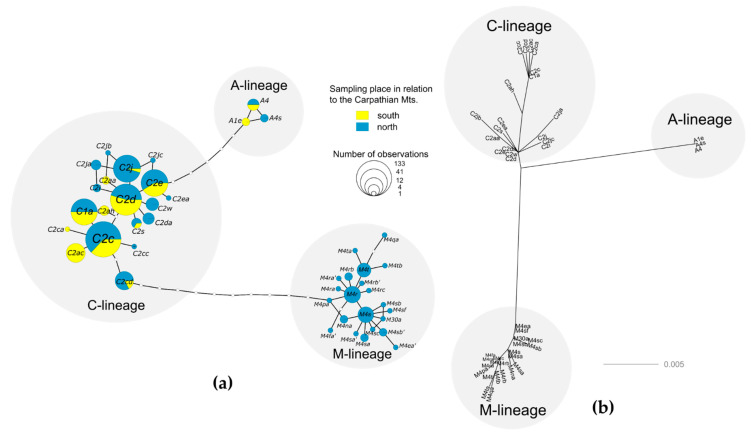
(**a**) The haplotype network of the COI-COII region in the mtDNA, based on a statistical parsimony with a 95% connection limit and simple indel coding method [56]. The area of a circle is proportional to the logarithm of the number of observed individuals. (**b**) Unrooted tree of 45 unique COI-COII sequences, inferred by using the Maximum Likelihood method and Hasegawa–Kishino–Yano model [58]. The tree with the highest log likelihood (−1348.20) is shown. A discrete Gamma distribution was used to model evolutionary rate differences among sites (5 categories (+G, parameter = 0.2079)). The rate variation model allowed for some sites to be evolutionarily invariable ([+I], 67.94% sites). The tree is drawn to scale, with branch lengths measured in the number of substitutions per site. Codon positions included were 1st + 2nd + 3rd + Noncoding. There were a total of 1042 positions in the final dataset. Evolutionary analyses were conducted in MEGA X [57].

We successfully amplified and obtained sequences from 444 samples. Seventeen samples were rejected by microsatellite relatedness analysis, as workers could be mothered by same queen, resulting in 427 workers for further examination. In these studied samples, we identified 45 unique mitotypes (Figure 2 and Figure 3, Supplementary Material S1 and S2). The detected mitotypes could be grouped into three evolutionary lineages, depending on the presence and number of repeat motifs P, P_0_ and Q [49,50]: the Southeast European C-lineage (Q, *N* = 376); the Western and Northern European M-lineage (PQ, *N* = 1; PQQ, *N* = 36; PQQQ, *N* = 7); and the African A-lineage (P_0_Q, *N* = 2; P_0_QQ, *N* = 5) (Table 1, Figure 3).

The most abundant C-mitotypes accounted for 88.06% of all mitotypes (with 95% bootstrap CIs from 84.60% to 90.98%), while M-mitotypes were significantly less numerous (10.30%, 95% CIs from 7.59% to 13.59%) and occurred only north of the Carpathians (Figure 1c). The frequency of M-mitotypes decreased from north to south, while C-mitotypes showed the opposite trend (Figure 1). Moreover, logistic regression (Table 2) indicated that latitude was a significant predictor of M mitotype presence (*p* < 0.001). M-mitotypes were not present to the south of the Carpathians, and they occurred slightly more often in eastern Poland, where they also ranged further south (Figure 1c). The categorical west/east variable ‘transect’, however, did not prove to be a highly significant predictor for M mitotype presence (*p* = 0.08).

African mitotypes were found in 1.64% of studied bees (95% CIs from 0.66% to 3.35%). Bees bearing A mitotypes were detected in Romania (n = 1), Hungary (n = 3) and Poland (n = 3), without a statistically significant spatial tendency (Figure 1b,e).

## 4. Discussion

Our results show for the first time that almost 2% of honey bees in East-Central Europe have mitochondrial DNA of African origin. As far as we know, this type of introgression has only previously been reported in Europe in the southern-western Iberian Peninsula [30,31,32,33,34,35], Sicily [37,61], the Balearic Islands [39], Malta [38] and France [36,62].

The presence of African mitotypes in the Iberian Peninsula and Mediterranean Islands can be explained by natural gene flow due to the similarity of their current climates and the existence of land bridges connecting these regions during ice ages [30,31,32,33,34]. In France, on the other hand, African mitotypes were probably introduced by humans, as in 1990s studies of the region, these haplotypes were not detected [31,51]. Moreover, haplotype A4, which is recorded in France, has not been identified in Spain [33,34].

The presence of African haplotypes in East-Central Europe is more likely to be caused by trading. In earlier studies, no African haplotypes were found in Europe to the north and east of France [8,9,11,63]. Neither of the three African haplotypes reported here (A1e, A4 and A4s) were recorded in Iberian Peninsula [32,33,35]. Moreover, haplotype A2, which is most common in Spain [34], was not recorded in this study. Two of the African haplotypes reported here (A4, A1e) were recorded previously in Africa [64] and in Africanized bees in North America [65] and South America [66]. The third African haplotype (A4s) was most similar to one recorded again in South America [66]. Thus, the likely origin of the A-lineage bees found in our study is Sub-Saharan Africa. It is also possible that genetic material was first introduced to the Americas and later from there to Europe.

In Eastern Europe, subspecies from lineages M and C are separated from African subspecies by subspecies from lineage O [22,67]. Moreover, there is no clear pattern in the geographic distribution of the African mitotypes in the study area. Therefore, it is unlikely that natural processes caused the presence of African mitotypes in Eastern and Central Europe. It is more likely that the presence of African mitotypes in this region is related to human activities. In a few cases, African subspecies were imported to Europe for experimental purposes [68,69,70,71]. However, as far as we know, the number of colonies imported for this purpose was small and they did not survive very well [71]; therefore, it is unlikely that the African haplotypes observed here originated from those bees.

The African haplotype introgression observed in this study was most likely caused by beekeeping practices, including importation of non-native subspecies and their intensive breeding by honey bee queen breeders. Some of these breeding lines, in particular breeding lines referred to as “Buckfast” can be hybrids containing genes of African ancestry. It is generally assumed that Buckfast bees represent C-lineage [72,73,74]. However, some reports reveal that two African subspecies, *A. m. sahariensis* and *A. m. monticola*, were used in crosses to obtain the Buckfast line [75,76,77]. It has been confirmed that more than 2% of Buckfast bees contain African haplotype A1 [78]. Both *A. m. sahariensis* and *A. m. monticola* contain haplotypes A1 and A4 [51] which were recorded in this study. However, it is worth noting that Buckfast bees can be very heterogeneous and the share of African ancestry can vary greatly, as there is no central supervision over the breeding program of this strain–any beekeeper can produce hybrids and distribute them as “Buckfast bees”. The increasing popularity of Buckfast bees has been reported in Slovakia [79] and Great Britain [80], and they have also been introduced in other European countries including Poland and Hungary [81].

So far, most of the growing concern regarding the conservation of bee genetic resources in Europe has been focused on *A. m. mellifera* [8,9,11,82]. The predominant subspecies of the evolutionary C-lineage, especially the Italian *A. m. ligustica* and Carniolan bee *A. m. carnica*, which are strongly preferred by beekeepers, have seemed less vulnerable, and in some cases, were even perceived as a threat to other subspecies or local ecotypes of honey bees [61,83,84]. Our study, which was carried out in the northern part of the native range of the Carniolan bee, shows that this species, highly appreciated by beekeepers, may also be threatened by hybridisation with bees from A-lineage.

Bees of African origin may pose a threat, not only to genetic diversity, but also to beekeeping and public health in Europe. They are less suitable for beekeeping, because of their propensity for absconding [85]. Moreover, one of the African subspecies, *A. m. capensis,* can produce females from unfertilized eggs, and clones of this subspecies have caused substantial losses for the beekeeping industry in South Africa [86]. African subspecies host parasites and pathogens which are not present in most of Europe, including small hive beetle [87]. Although the imported queens are screened for diseases, some as-yet-undescribed pathogens, for example, viruses, may go undetected. In general, it is believed that Africanized bees have caused significant problems related to beekeeping in the New World ([88,89,90,91], but see [92]).

The importation of African subspecies also poses a risk to public health. It is well known that Africanised bees are more defensive in comparison to European subspecies [93]; hence, they pose a higher risk of stinging incidents and related health issues among members of the general public [94].

African honey bee subspecies have proved to be invasive in the Americas [95,96,97]. Therefore, the USA and some other countries have introduced certification procedures to detect and control the spread of Africanized bees [98,99]. In consequence, no African mitotypes have been detected in queen breeding populations from the USA [73]. As far as we know, such control was not implemented in the European Union, where it is permitted to import queens from some non-EU countries [80]. Only the health of the bees, and not their genetic origin, is verified (Commission Regulation (EU) 206/2010). Among other origins, honey bee queens are imported to the EU from Argentina [42] where Africanized bees are present [100].

It can be argued that the climate in Europe is not suitable for Africanized bees. However, some other countries with similar or colder climates have banned the import of Africanized bees [101]. For example, in Canada, imported queens need a certificate to state that they are not Africanized [102]. Moreover, a warming climate may increase the likelihood of African hybridization in Europe [103].

From an economics perspective, Africanized bees have some advantages over European subspecies, as they tolerate parasitic mite-*Varroa destructor* [104,105,106,107]. This tolerance can be related to both a higher resistance of bees and a lower virulence of mites [108]. It was demonstrated that tolerance could depend on the environment [87,109]. Therefore, it is possible that varroa-tolerance of Africanized bees will be less effective in the European environment. Moreover, colonies tolerating varroa are also present in Europe [110]. In general, varroa-tolerant bees, either of African or European origin, are not used on a large scale because they are less suitable for beekeeping, especially as the mite tolerance mechanism is usually due to smaller colony size and a greater tendency to swarm [110].

It should be stressed that African hybridization was detected here using mitochondrial DNA. It remains to be clarified what the proportion of African genes in the nuclear genome is. Even if the nuclear genes are largely of European origin, the presence of African mitotypes indicates a large scale import of non-native subspecies, which needs to be monitored.

## 5. Conclusions

The presence of African mitotypes in honey bees from East-Central Europe deserves more attention, as it may contribute to the dissemination of undesirable traits, parasites and diseases. Importation and spread of alien honey bees should be better monitored as this could be a serious threat to the sustainable apiculture.

## Figures and Tables

**Table 1:** The proportion of honeybees carrying mitotypes of the three evolutionary lineages: south-eastern European (C), African (A) and West and North European (M), estimated based on total sample of 427 individuals.

Lineage	Sequence Type	Estimated Frequency (95% Confidence Intervals)
C	Q	0.881 (0.846–0.910)
	all C	0.881 (0.846–0.910)
A	P_0_Q	0.005 (0.001–0.017)
	P_0_QQ	0.012 (0.004–0.027)
	all A	0.016 (0.007–0.033)
M	PQ	0.002 (0.000–0.013)
	PQQ	0.084 (0.060–0.115)
	PQQQ	0.016 (0.007–0.033)
	all M	0.103 (0.076–0.136)

**Table 2:** Results of logistic regression models between mitotypes and location. Dependent variables (mitotypes from C-, A- and M-lineages) were coded as binary variables (yes or no). Transect was a factor variable with two levels (western vs. eastern). Values indicate regression coefficients and their standard errors are shown in brackets.

	C-Lineage	A-Lineage	M-Lineage
Latitude	−0.383 ***	−0.044	0.535 ***
	(0.079)	(0.136)	(0.108)
Transect ^#^	0.340	0.962	−0.615 ^+^
	(0.319)	(0.852)	(0.356)
Constant	21.578 ***	−2.441	−29.638 ***
	(4.155)	(6.947)	(5.681)
Model fit *χ*^2^ (2)	35.48 ***	1.68	48.17 ***
Pseudo-*R*^2^ (McFadden)	0.114	0.024	0.170

^#^ western in relation to eastern transect, *** *p* < 0.001, ^+^ 0.05 < *p* < 0.1.

## Data Availability

Data were submitted to the NCBI Nucleotide database at https://www.ncbi.nlm.nih.gov/nuccore/, accessed on 15 April 2021.

## Supplementary Materials

The following are available online at https://www.mdpi.com/article/10.3390/insects12050410/s1, Table S1: Honeybee mitotypes found in this study, File S2: Aligned sequences in FASTA format.
