# Supplementary material for: Mitochondrial DNA Suggests the Introduction of Honeybees of African Ancestry to East-Central Europe
Source: Insects. 2021 May 2;12(5):410. doi: 10.3390/insects12050410 (PMC8147603; doi:10.3390/insects12050410)
Supplement: Supplementary file 1 [file insects-12-00410-s001.zip › Table_S1.pdf]

Table S1. Honeybee mitotypes found in East-Central Europe.

Mitotype	Lineage	Sequence type	Number of samples	Estimated proportion (95% confidence intervals)	Accession number	Names of the most similar mitotypes present in the GenBank – accession number (% similarity)
**C2d**	C	Q	75	0.176 (0.141, 0.215)	MW939577	C2d - MF136776 (100)<br>C2d - JF723977 (100)<br>C12 - FJ037777 (100)
**C2i**	C	Q	2	0.005 (0.001, 0.017)	MW939606	C2i - JQ977703 (100)<br>C19 - FJ037782 (100)
**C2aa**	C	Q	2	0.005 (0.001, 0.017)	MW939619	C2aa - MG788257 (100)
**C2j**	C	Q	39	0.091 (0.066, 0.123)	MW939580	C2j - JF723978 (100)<br>C11 - FJ037776 (100)
**C2ja**	C	Q	3	0.007 (0.001, 0.02)	MW939587	C2i - JQ977703 (99.82)<br>C2j - JF723978 (99.82)
**C2jb**	C	Q	1	0.002 (0, 0.013)	MW939592	*A. m. caucasica* mitochondrion, complete genome, unnamed mitotype - MN714160 (100)<br>C2j - MH939345 (99.82)<br>C11 - FJ037776 (99.82)
**C2s**	C	Q	3	0.007 (0.001, 0.02)	MW939585	C2s - JF723979 (100)<br>C31 - HQ287900 (100)
**C2c**	C	Q	133	0.309 (0.268, 0.358)	MW939584	C2c - JF723976 (100)<br>C2c - HQ199227 (100)
**C2cc**	C	Q	1	0.002 (0, 0.013)	MW939600	C2v1 MH939336 (99.82)<br>C2c - JF723976 (99.82)<br>C2c - HQ199227 (99.82)
**C2ca**	C	Q	1	0.002 (0, 0.013)	MW939618	C2c - JF723976 (99.82)<br>C2c - HQ199227 (99.82)
**C2ac**	C	Q	11	0.026 (0.013, 0.046)	MW939620	C2ac - MT741501 (100)
**C2ah**	C	Q	3	0.007 (0.001, 0.02)	MW939617	C2ah - MT741506 (100)
**C2w**	C	Q	5	0.012 (0.004, 0.027)	MW939578	C2w - MT741499 (100)
**C2jc**	C	Q	1	0.002 (0, 0.013)	MW939589	C2j - MH939345 (99.82)<br>C2e - JQ977702 (99.82)<br>C11 - FJ037776 (99.82)
**C2e**	C	Q	41	0.096 (0.07, 0.128)	MW939590	C2e - JQ977702 (100)
**C2ea**	C	Q	1	0.002 (0, 0.013)	MW939607	C2e - JQ977702 (99.82)
**C2da**	C	Q	4	0.009 (0.003, 0.024)	MW939579	C2d - JF723977 (99.30)

**Mitotype**	**Lineage**	**Sequence type**	**Number of samples**	**Estimated proportion (95% confidence intervals)**	**Accession number**	**Names of the most similar mitotypes present in the GenBank – accession number (% similarity)**
						C27 - HQ287899 (99.30)<br>C12 - FJ037777 (99.30)
**C1a**	C	Q	38	0.089 (0.064, 0.12)	MW939583	C1a - JQ977699 (100)<br>C1 - MF136775 (100)
**C2cd**	C	Q	12	0.028 (0.015, 0.049)	MW939593	*A. m. carpathica* “Sklenar 47#4”, unnamed mitotype - MK140902 (100)<br>*A. m. carpathica* “Hoverla”, unnamed mitotype – MK140896 (99.82)<br>C2c - JF723976 (99.82)<br>C1a - JQ977699 (99.82)
**A1e**	A	P_0Q	2	0.005 (0.001, 0.017)	MW939582	A1e - GU326335 (100)<br>*A. m. capensis*, unnamed haplotype - MG552696 (100)<br>*A. m. scutellata*, unnamed haplotype - KY614238
**A4s**	A	P_0QQ	2	0.005 (0.001, 0.017)	MW939597	A4, Africanised honeybee from Brazil - EF033650 (99.88)<br>A4-BEN4, *A. m. adansonii* from Benin - MG592303 (99.76)<br>A26d, Africanised honeybee from USA - GU326336 (99.76)
**A4**	A	P_0QQ	3	0.007 (0.001, 0.02)	MW939614	A4 - EF033650 (100)
**M4r**	M	PQQ	9	0.021 (0.01, 0.04)	MW939581	*A. m. sinisxinyuan*, unnamed mitotype - MN733955 (99.76)<br>M4 - EF033656 (99.76)<br>M4q - MK387716 (99.76)
**M4t**	M	PQQ	6	0.014 (0.005, 0.03)	MW939586	M4 - EF033656 (99.88)
**M4ta**	M	PQQ	1	0.002 (0, 0.013)	MW939611	M4 - EF033656 (99.76)
**M4pa**	M	PQQ	1	0.002 (0, 0.013)	MW939604	M4p - KX463897 (99.64)<br>M4m - KX463894 (99.64)<br>M17i - KX463898 (99.64)
**M4na**	M	PQQ	2	0.005 (0.001, 0.017)	MW939588	M4n - KX463895 (99.76)<br>*A. m. sinisxinyuan*, unnamed mitotype - MN733955 (99.76)
**M4s**	M	PQQ	8	0.019 (0.008, 0.037)	MW939595	*A. m. sinisxinyuan*, unnamed mitotype - MN733955 (99.88)<br>M4n - KX463895 (99.64)<br>M4q - MK387716 (99.64)
**M4sa**	M	PQQ	1	0.002 (0, 0.013)	MW939596	*A. m. sinisxinyuan*, unnamed mitotype - MN733955 (99.76)<br>M4n - KX463895 (99.52)

Mitotype	Lineage	Sequence type	Number of samples	Estimated proportion (95% confidence intervals)	Accession number	Names of the most similar mitotypes present in the GenBank – accession number (% similarity)
						M4q - MK387716 (99.52)
**M4sb**	M	PQQ	1	0.002 (0, 0.013)	MW939598	*A. m. sinisxinyuan*, unnamed mitotype - MN733955 (99.76)<br>M4q - MK387716 (99.52)<br>M4n - KX463895 (99.52)<br>M4 - EF033656 (99.52)
**M4ra**	M	PQQ	1	0.002 (0, 0.013)	MW939603	M4q - MK387716 (99.76)<br>*A. m. sinisxinyuan*, unnamed mitotype - MN733955 (99.64)
**M4tb**	M	PQQ	1	0.002 (0, 0.013)	MW939605	M77’ - KX463940.1 (99.45)
**M4rc**	M	PQQ	1	0.002 (0, 0.013)	MW939610	*A. m. sinisxinyuan*, unnamed mitotype - MN733955 (99.52)<br>M4q - MK387716 (99.52)
**M4rb**	M	PQQ	1	0.002 (0, 0.013)	MW939612	*A. m. sinisxinyuan*, unnamed mitotype - MN733955 (99.88)<br>M4q - MK387716 (99.64)
**M4ra'**	M	PQQQ	1	0.002 (0, 0.013)	MW939615	M77' - KX463940 (98.83)<br>M4f' - KX463934 (98.83)<br>M4e' - KX463933 (98.83)
**M4sa'**	M	PQQQ	1	0.002 (0, 0.013)	MW939591	M77' - KX463940 (99.41)<br>M4f' - KX463934 (99.41)<br>M4e' - KX463933 (99.41)
**M4ea'**	M	PQQQ	1	0.002 (0, 0.013)	MW939601	M77' - KX463940 (99.32)<br>M4f' - KX463934 (99.32)<br>M4e' - KX463933 (99.32)
**M4sb'**	M	PQQQ	2	0.005 (0.001, 0.017)	MW939602	M77' - KX463940 (99.41)<br>M4f' - KX463934 (99.41)<br>M4e' - KX463933 (99.41)
**M4fa'**	M	PQQQ	1	0.002 (0, 0.013)	MW939594	M77' - KX463940 (99.71)<br>M4f' - KX463934 (99.71)<br>M4e' - KX463933 (99.71)
**M4sf**	M	PQQ	1	0.002 (0, 0.013)	MW939609	*A. m. sinisxinyuan*, unnamed mitotype - MN733955 (99.76)<br>M4q - MK387716 (99.52)<br>M4n - KX463895 (99.52)<br>M5 - FJ743638 (99.52)
**M4sc**	M	PQQ	1	0.002 (0, 0.013)	MW939608	*A. m. sinisxinyuan*, unnamed mitotype - MN733955 (99.76)<br>M4q - MK387716 (99.52)<br>M4n - KX463895 (99.52)

**Mitotype**	**Lineage**	**Sequence type**	**Number of samples**	**Estimated proportion (95% confidence intervals)**	**Accession number**	**Names of the most similar mitotypes present in the GenBank – accession number (% similarity)**
**M4qa**	M	PQQ	1	0.002 (0, 0.013)	MW939616	M4 - EF033656 (99.64)<br>*A. m. sinisxinyuan*, unnamed mitotype - EF033656 (99.64)<br>M4q - MK387716 (99.39)
**M4rb'**	M	PQQQ	1	0.002 (0, 0.013)	MW939613	M77' - KX463940 (99.41)<br>M4f' - KX463934 (99.41)<br>M4e' - KX463933 (99.41)
**M30a**	M	PQ	1	0.002 (0, 0.013)	MW939599	*A. m.* unnamed mitotype -KT164631 (99.68)<br>(draI restriction pattern similar to mitotype M30, HQ260340)
